# ADC values as a biomarker of fetal brain maturation

**DOI:** 10.2478/raon-2023-0022

**Published:** 2023-06-21

**Authors:** Lucija Kobal, Katarina Surlan Popovic, Jernej Avsenik, Tina Vipotnik Vesnaver

**Affiliations:** Faculty of Medicine, University of Ljubljana, Ljubljana, Slovenia; Clinical Institute of Radiology, University Medical Centre Ljubljana, Ljubljana, Slovenia

**Keywords:** myelination, fetal brain maturation, ADC, biomarker, diffusion-weighted imaging, diffusion

## Abstract

**Background:**

During the period of fetal development, myelination plays a key role and follows specific time and spatial sequences. The water content in the brain is inversely proportional to myelination – the more myelinated the brain, the lower the water content in it. The diffusion of water molecules can be quantitatively assessed using the apparent diffusion coefficient (ADC). We were interested in whether, by determining the ADC values, we could quantitatively evaluate the development of the fetal brain.

**Patients and methods:**

The study included 42 fetuses with gestational age 25 to 35 weeks. We manually selected 13 regions on diffusion-weighted images. Statistically significant differences between ADC values were checked using one-way analysis of variance and Tukey's post hoc test. The relationship between the ADC values and the gestational age of the fetuses was then assessed using linear regression.

**Results:**

The average gestational age of the fetuses was 29.8 ± 2.4 weeks. ADC values in the thalami, pons and cerebellum differed significantly among each other and from the ADC values in other brain regions. In the thalami, pons and cerebellum, linear regression showed a significant decrease in ADC values with increasing gestational age.

**Conclusions:**

ADC values change with the increasing gestational age of the fetus and differ among different brain regions. In the pons, cerebellum and thalami, the ADC coefficient could be used as a biomarker of fetal brain maturation since ADC values decrease linearly with increasing gestational age.

## Introduction

Human brain development is a complex process that begins in the third gestational week and continues well after birth, even into adulthood.^1^ In our study, we were interested in the process of myelination and how it can affect apparent diffusion coefficient (ADC) values.

Myelination is the final stage in the development of the white matter of the brain. It begins in the second half of pregnancy, after the proliferation and maturation of oligodendrocyte cells. It also has a characteristic course, from caudal to rostral regions of the brain, from the center outwards, and from dorsal to ventral regions of the brain.^2,3^ As early as around the 20^th^ week of gestation, microscopic amounts of myelin can be observed, especially in the medulla oblongata and pons. The brainstem is completely myelinated at 29 weeks.^4^ Between the 37^th^ and 40^th^ gestational week, mature myelin is present in the cerebellum and the internal capsule.^4,5^ Sensory pathways are myelinated earlier than motor pathways, and occipital areas are myelinated earlier than parietal, temporal and frontal areas. Myelination of the brain also takes place in the postnatal period – it is completed only after the age of 20 when the areas of the corpus callosum and prefrontal brain become fully myelinated.^6,7,8,9^

Knowing the exact course of myelination is crucial for detecting pathological changes that affect myelination. In premature infants, hypomyelination may be a predictive factor for motor and cognitive impairments. Myelination can also be affected by many genetic and autoimmune diseases and infections.^10,11,12^

As the brain matures, the water content changes over time. The amount of water in the brain is inversely proportional to myelination - the more myelinated the brain, the less water it contains. The decrease in water content can be attributed to the accumulation of lipids and proteins and changes in the electrolytic composition of tissues. The reduction in the proportion of water continues even after birth. In addition to the water content, the movement of water molecules in the brain is also affected by the number of cells and the amount of myelin, both of which limit the movement of molecules.^13,14,15,16,17^

With the development of MRI, especially with the advance of diffusion-weighted magnetic resonance imaging (DWI), an opportunity has appeared for the non-invasive assessment of myelination in fetuses. Furthermore, the diffusion of water molecules can now be quantitatively evaluated using the ADC values.

DWI is an extremely useful technique for detecting hyperacute hypoxic-ischemic changes, it can be helpful in other disease processes that affect the movement of water molecules in tissues, *e.g.*, abscesses, tumours etc. and it can also be used to monitor the normal development of the fetal brain.^18,19,20,21,22,23^ This technique is already a part of the standard protocol in fetal MR imaging to diagnose anomalies of the central nervous system but, despite previous studies that have addressed this issue, it is still not completely clear whether a quantitative evaluation of fetal brain development using ADC values can be used in clinical practice. We do not yet have reference ADC values that would allow us to compare healthy fetuses with fetuses suspected of developing anomalies of the central nervous system.^24,25,26,27,28,29,30^ Research is therefore increasingly focused on studying ADC values in different areas of the fetal brain.

In our study, we observed how ADC values change during the process of fetal brain maturation. We postulated that the values would depend on the gestational age of the fetus and also on the areas of the brain. The determined ADC values could serve as reference values in daily clinical work. They would be useful in assessing the level of fetal brain maturity and early detection of pathological changes.

The purpose of our research was to determine how ADC values change with the gestational age of the fetus. We were interested in how ADC values differ among different brain areas. We also wanted to discover whether the ADC could be used as a biomarker of fetal brain maturation.

Our hypotheses were:

1. ADC is a useful biomarker of fetal brain maturation.
2. ADC values depend on the age of the fetus and the area of the brain.

## Patients and methods

The retrospective study was conducted at the Clinical Institute of Radiology of the University Medical Center Ljubljana. The National Medical Ethics Committee of the Republic of Slovenia judged that the research was ethically acceptable and gave consent for its implementation (No. 0120-56/2022/3).

### Patients

We initially selected 59 fetuses that had had an MRI done between 18. 1. 2015 and 4. 3. 2021. Of these, the MRI images of 17 fetuses were excluded since their DWI images had artefacts due to fetal movement. We therefore performed the measurements on MRI images of 42 fetuses. We included fetuses that have been referred for MRI due to suspicious US changes in the central nervous system (CNS), face or neck (wider cisterna magna, suspected agenesis of the corpus callosum, ranula etc.) but in which we did not confirm CNS anomalies with MRI. All included pregnant women underwent amniocentesis, which excluded chromosomal abnormalities and infections. After birth, no signs indicating abnormal development of the central nervous system were found in our group. The gestational age of the fetuses was from 25 to 35 weeks (mean 29.8 ± 2.4 weeks).

### Magnetic resonance imaging

Examinations were performed on a Siemens Aera 1.5 T MR device. The pregnant women did not eat or drink for four hours before the examination and, during the examination, they were in a supine or left decubitus position. They did not receive any medication before the procedure. An abdominal coil with a small, 24 cm field of view and a 192 × 160 matrix was used. A 3D scout sequence was performed to assess the fetal position. Scout sequence is an ultrafast T2-weighted sequence, with slice thicknesses of 6–8 mm, gaps of 1–2 mm, and a large field of view. Our protocol consisted of ultra-fast T2-weighted sequences in three planes (axial, coronary, sagittal) with slice thicknesses of 3 and 4 mm and intermediate intervals of 0.3 mm, T1- and T2-gradient sequences in the axial plane (the former with slice thickness 4.5 mm and intermediate intervals 0.5 mm, the latter with slice thickness 3 mm and intermediate intervals 0.3 mm) and DWI-sequence in the axial plane (slice thickness 5 mm with intermediate intervals 2 mm). Diffusion was measured in three directions at values of b = 0 s/mm^2^, b = 500 s/mm^2^, and b = 1000 s/mm^2^.

### ADC value measurements

ADC measurements were performed by two researchers (L.K., T.V.V.). On ADC maps, we manually selected 13 areas (region of interest, ROI) using a Syngo. via program (Siemens Medical Solutions USA, Inc. ©2022): bilaterally in the frontal white matter (FWM), parietal white matter (PWM), temporal white matter (TWM) and occipital white matter (OWM), bilaterally in the white matter of the cerebellum, in both thalami and the central part of the pons. The surface area of the ROI was adapted to the age of the fetus and the anatomical area, with values between 15 and 65 mm^2^.

**FIGURE 1.**
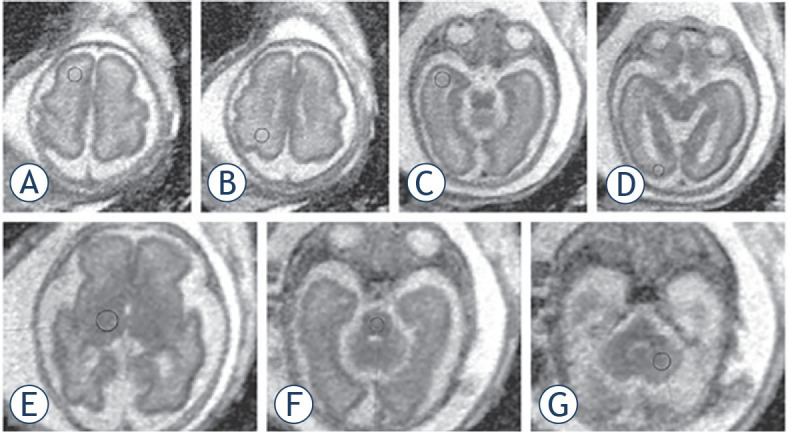
Position of the ROI in the brain of a fetus with a gestational age of 29 weeks. Region of interests (ROIs) were placed in each brain region bilaterally. Markings are visible only on one side. **(A)** Frontal white matter, **(B)** parietal white matter, **(C)** temporal white matter, **(D)** occipital white matter, **(E)** thalamus, **(F)** pons, **(G)** hemisphere of the cerebellum.

### Statistical data analysis

Statistical data analysis and graph production were performed using IBM SPSS Statistics (IBM SPSS Statistics for Windows, version 25.0; IBM Corp., Armonk, NY). Interrater reliability was assessed using the intraclass correlation coefficient (ICC) with a two-way mixed model for the average of the measurements.

ICC values below 0.5 indicate low, between 0.5 and 0.75 moderate, between 0.75 and 0.9 good, and above 0.9 excellent reliability. Since the ICC was 0.75 or higher in all measured brain areas, we averaged the measurements of the two researchers. Statistically significant differences between ADC values for different areas of the brain were checked using one-way analysis of variance (ANOVA). Statistically significant differences between ADC values in individual groups were searched for with Tukey's post hoc test. Before the one-way analysis of variance, we checked the homogeneity of the variances with Levene's test, whereby we rejected the null hypothesis that the variances are homogeneous or homoscedastic. The association between the ADC values of the brain regions and the gestational age of the fetuses was then evaluated using linear regression. Before the statistical data analysis, we determined the p-value 0.05 as the threshold.

## Results

ICC values by individual brain areas are shown in Table 1.

**TABLE 1.** Intraclass correlation coefficient (ICC) for different regions of interest (ROIs)

**ROI**	**ICC**
FWM	0.91
PWM	0.90
TWM	0.75
OWM	0.78
Thalami	0.85
Pons	0.81
Cerebellum	0.92

FWM = frontal white matter; OWM = occipital white matter; PWM = parietal white matter; TWM = temporal white matter

**FIGURE 2.**
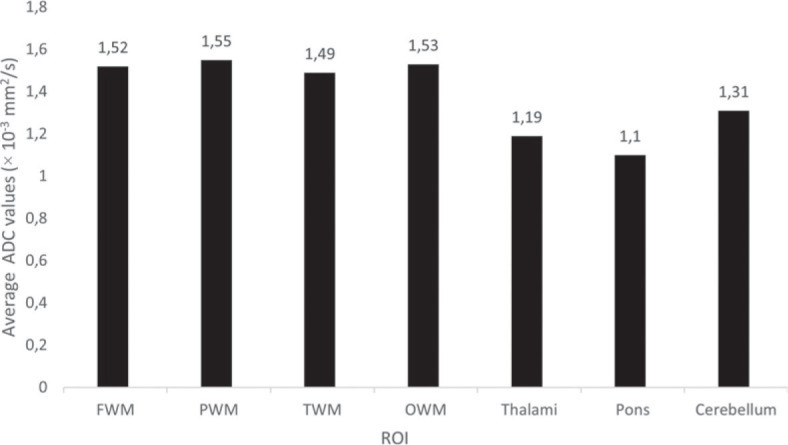
Average apparent diffusion coefficient (ADC) values for different regions of interest (ROIs).

The average ADC values for different ROIs are shown in Figure 2. Using one-way analysis of variance, we found that there was a statistically significant difference between the ADC values for different brain areas. The ADC values in the thalami, pons and cerebellum differed significantly from each other and from the ADC values in all other brain areas. There were no significant differences between ADC values in FWM, PWM, TWM and OWM.

**FIGURE 3.**
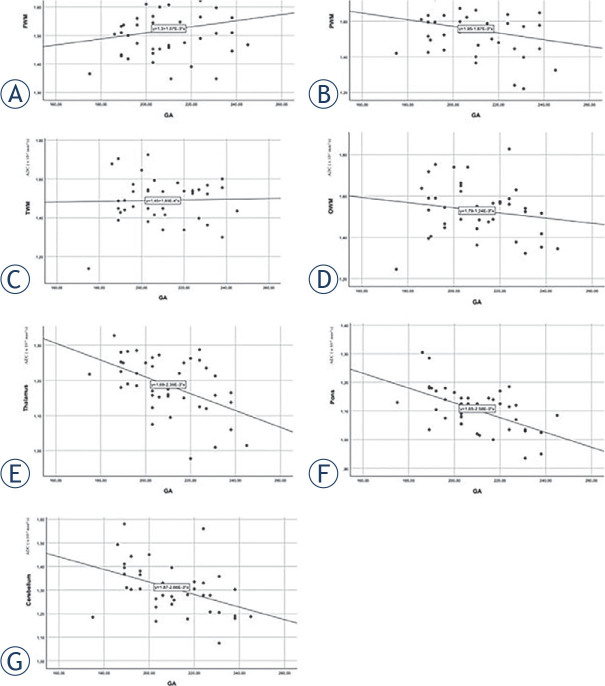
Association between apparent diffusion coefficient (ADC) values in different brain regions and fetal gestational age in days. **(A)** Frontal white matter, **(B)** parietal white matter, **(C)** temporal white matter, **(D)** occipital white matter, **(E)** thalamus, **(F)** pons, **(G)** cerebellum.

In the thalami, pons and cerebellum, linear regression showed a statistically significant decrease in ADC values with increasing gestational age (Figure 3). ADC values also decreased with increasing gestational age in PWM and OWM, but the results were not statistically significant. ADC values increased in FWM and TWM, but the increase in values in these areas was also not statistically significant.

## Discussion

Our study confirmed that the average ADC values in fetuses differ among different brain areas. As shown in Figure 2, the lowest values were measured in the pons, thalami and cerebellum, and the highest in the FWM, PWM, TWM and OWM. These differences can be attributed to the characteristic course of myelination and the number of neurons in individual areas. The amount of neurons in the thalami is greater than in the white matter, so the diffusion of water is more limited. Since myelination proceeds from the caudal to the rostral regions of the brain, from the central regions to the periphery, and from the dorsal to the ventral regions, the pons, thalami and cerebellum are myelinated earlier than the white matter of the cerebral hemispheres, where the myelination process still takes place in the postnatal period. ADC values in fetuses, therefore cannot be compared with values in newborns and children, nor with values in preterm infants of the same gestational age, since large changes in the amount and distribution of water in the brain occur after birth.^2,3^

ADC values in the pons, thalami and cerebellum were much lower than in the white matter of the cerebral hemispheres, which is consistent with previous research. The average values in these areas were comparable with previous studies, while the average ADC values for FWM, PWM, TWM and OWM were slightly lower in our study. The largest deviations can be seen for the FWM area, where in our research we measured an average ADC value of 1.52 × 10^−3^ mm^2^/s. Han *et al.* (1.8 × 10^−3^ mm^2^/s), Schneider *et al.* (1.8 × 10^−3^ mm^2^/s), Hoffmann *et al.* (1.8 × 10^−3^ mm^2^/s) and Righini *et al.* (2.9 × 10^−3^ mm^2^/s) all recorded higher values.^25,27,28,29,30,31^ These differences could be attributed to the slightly lower average age of the fetuses in our study and the possible non-linear changing of ADC values, as described below.

OWM is known to be myelinated earlier than PWM, TWM and FWM.^8^ We were unable to confirm this with our research. The results of our research showed that ADC values did not differ statistically significantly among these areas. We attribute these results to the fact that the occipital part of the brain in fetuses is thinner than in the rest of the cerebral hemispheres. The ROIs were therefore placed closer to the cerebrospinal fluid than in other areas, which may have resulted in partial coverage of the cerebrospinal fluid signal in the ROI. As a result, measurements may be affected by averaging the values of cerebrospinal fluid and brain parenchyma. This may also explain why the ICCs between the researchers differed predominantly in the areas of OWM and TWM, where the cerebral mantle is also thinner.

In the thalami, pons and cerebellum, linear regression showed a statistically significant decrease in ADC values with increasing gestational age (Figure 3). ADC values also decreased with increasing gestational age in PWM and OWM, but the results were not statistically significant. In FWM and TWM, ADC values increased in our study, but the increase in values was not statistically significant in these areas either. The FWM is the last of all brain areas to be myelinated, the amount of water there being the highest for the longest time, which could be the reason for the measured values in our research.

All the studies published so far have confirmed the decline in ADC values with increasing gestational age in the areas of the cerebellum, thalami and pons.^25,27,28,29,30,31^ The results indicate that in these areas of the brain, the amount of water decreases with age, which affects the reduction of ADC values. In our study, ADC values also declined most rapidly in the cerebellum, pons and thalami, which suggests earlier maturation of these regions and is consistent with other studies.

Differences among studies occurred in other brain areas. A decrease in ADC values in PWM and OWM was demonstrated by Han *et al.*, Hoffmann *et al.* and Righini *et al.*, while Cannie *et al.* detected an increase in ADC values in OWM.^27,28,29,30^ Similar to our study, some other researchers have also detected an increase in ADC values with gestational age in FWM.^48,50,53^ Schneider *et al*, on the other hand, measured increasing ADC values in FWM, PWM, TWM and OWM until the 30^th^ gestational week, but thereafter the ADC values began to decrease.^31^

The results of Schneider *et al.* are consistent with the course of fetal brain development. During development, the brain consists of several layers - above the ventricular layer, there is an intermediate layer, a subplate and a cortical layer. Wide extracellular spaces are present in the intermediate layer and subplate, which allow nerve cells to migrate. In these spaces, water molecules can move freely, which could explain the rise in ADC values. Schneider *et al.* explained the repeated decline in ADC value by a combination of different factors that begin to dominate after the 30^th^ week: the subplate and the intermediate layer slowly disappear, the total amount of water decreases and, at the same time, the number of lipids and macromolecules in the intracellular spaces increases. We therefore allow the possibility that the change in ADC values in FWM, PWM, TWM and OWM with increasing gestational age is not linear, but may be better explained by more complex models, for example using a quadratic polynomial curve, which means that the values initially increase and then decrease.^31^

The multilayered structure of the brain is visible on T2-weighted sequences in fetuses of the gestational age of 20–28 weeks but, with increasing gestational age, the boundaries between the layers are blurred. Due to the poor image resolution of the ADC maps, it is very difficult to distinguish the layers from each other, even in younger fetuses. When manually placing the ROIs, due to the poor resolution on the ADC maps we may also capture areas of the subplate, which later develop into the cerebral cortex, and not the white matter, which could affect the results of the measurements.

The downside of our research was the relatively small number of subjects. A disadvantage is also the probability of measurement errors due to the movement of the fetus during the examination, which was reduced by preparing the mothers for the MRI examination and eliminating poor-quality images. In fetal MRI, measurements are also affected by the small size of the structures, which may result in the capture of the cerebrospinal fluid or subplate in selected cases. ROI drawing may also be challenging due to the poor resolution of the brain layers on the ADC maps.

Another thing to consider before comparing results from different studies is variability in measured ADC values due to the use of different MRI equipment and sequence selections.

## Conclusions

We conclude that ADC values are a reliable indicator of brain maturation in the areas of the pons, cerebellum and thalami. The results of our research are concordant with previous research, which has shown that the values in these areas decrease linearly with increasing gestational age. We also confirmed that the ADC values are higher in the FWM, PWM, TWM, and OWM regions than in the pons, cerebellum and thalami. However, it is still unknown how ADC values in FWM, PWM, TWM and OWM change with gestational age, since results vary among studies. Further research is needed to define more precisely the variation of ADC values in these areas, which would help us to set reference values. Nevertheless, we confirmed our second hypothesis that ADC values in different brain areas differ from each other and also change over time.
